# Zwitterionic Polymer Brush Grafted on Polyvinylidene Difluoride Membrane Promoting Enhanced Ultrafiltration Performance with Augmented Antifouling Property

**DOI:** 10.3390/polym12061303

**Published:** 2020-06-07

**Authors:** Yu-Hsuan Chiao, Shu-Ting Chen, Mani Sivakumar, Micah Belle Marie Yap Ang, Tanmoy Patra, Jorge Almodovar, S. Ranil Wickramasinghe, Wei-Song Hung, Juin-Yih Lai

**Affiliations:** 1 Advanced Membrane Materials Research Center, Graduate Institute of Applied Science and Technology, National Taiwan University of Science and Technology, Taipei 10607, Taiwan; sc068@uark.edu (S.-T.C.); sivaphy05@gmail.com (M.S.); jylai@mail.ntust.edu.tw (J.-Y.L.); 2 Department of Chemical Engineering, University of Arkansas, Fayetteville, AR 72701, USA; jlalmodo@uark.edu (J.A.); swickram@uark.edu (S.R.W.); 3 R&D Center for Membrane Technology and Department of Chemical Engineering, Chung Yuan Christian University, Chung Li 32023, Taiwan; mbmyang@gmail.com; 4 Department of Biomedical Engineering, University of Arkansas, Fayetteville, AR 72701, USA; tpatra@uark.edu

**Keywords:** zwitterionic, PVDF membrane, poly (sulfobetaine methacrylate), ultrafiltration, antifouling, UV grafting

## Abstract

Superhydrophilic zwitterions on the membrane surface have been widely exploited to improve antifouling properties. However, the problematic formation of a <20 nm zwitterionic layer on the hydrophilic surface remains a challenge in wastewater treatment. In this work, we focused on the energy consumption and time control of polymerization and improved the strong hydrophilicity of the modified polyvinylidene difluoride (PVDF) membrane. The sulfobetaine methacrylate (SBMA) monomer was treated with UV-light through polymerization on the PVDF membrane at a variable time interval of 30 to 300 s to grow a poly-SBMA (PSBMA) chain and improve the membrane hydrophilicity. We examined the physiochemical properties of as-prepared PVDF and PVDF–PSBMA_x_ using numeric analytical tools. Then, the zwitterionic polymer with controlled performance was grafted onto the SBMA through UV-light treatment to improve its antifouling properties. The PVDF–PSBMA_120s_ modified membrane exhibited a greater flux rate and indicated bovine serum albumin (BSA) rejection performance. PVDF–PSBMA_120s_ and unmodified PVDF membranes were examined for their antifouling performance using up to three cycles dynamic test using BSA as foulant. The PVDF-modified PSBMA polymer improved the antifouling properties in this experiment. Overall, the resulting membrane demonstrated an enhancement in the hydrophilicity and permeability of the membrane and simultaneously augmented its antifouling properties.

## 1. Introduction

Membrane-based techniques have recently been broadly utilized in water purification and separation of contaminated wastewater due to their low energy consumption, excellent separation performance, reliability, space-saving efficiency, and environmental friendliness [1,2]. Unfortunately, the protein separation membrane used for filtration can be afflicted by several problems related to biological and organic fouling, which raises the operating costs. This problem is normally solved by improving the membrane surface hydrophilicity with the addition of the hydrophilic fabric, as this can provide strong protein resistance based on the steric hindrance and hydration shell [3,4,5]. Significant adsorption and aggregation of foulants on the membrane surface lead to a rapid critical reduction of separation performance, increased expenses for cleaning, and diminished membrane life. Thus, surface modification to membranes with hydrophilic materials has been necessary, and an effective method has been established to decrease interactions between the membrane and foulants to enable fouling resistance for the membranes [6]. Contrasted with polyvinylidene difluoride (PVDF) subordinates, zwitterionic materials form a more stable hydration layer than PVDF derivatives [7] and can oppose protein adsorption [6,8]. Due to ionic characters, zwitterionic synthetic substances attract more water molecules via hydrogen bonding and electrostatic interactions [9], which results in a thicker hydration layer than polyethylene glycol (PEG)-type chemicals [10]. The ability of zwitterionic synthetic concoctions to maintain a strategic distance from the adsorption of foulants onto the membrane was clarified as a consistently compacted water molecule [11] and lower hydration free energy than nonionic moieties [6,7]. The steric deterrent [12] and adversely charged surface additionally assume a significant role in zwitterionic materials [6]. Every one of these components contributes consistently to the antifouling qualities of zwitterionic chemicals in membranes.

PVDF membranes possess excellent chemical stability, mechanical strength, toughness and stiffness. Owing to these inherent properties, PVDF membranes have been widely applied in the membrane field, including air dehumidification [13], water purification [3,14,15], blood purification [16,17], organic solvent-resistant membranes [18], and gas separation [19]. However, their inherently strong hydrophobicity can lead to severe organic- and bio-fouling behavior to a greater extent than the other common polymeric materials, polyethersulfone (PES), polysulfone (PSF), polyacrylonitrile (PAN) and cellulose [20]. Furthermore, PAN and cellulose membranes have a narrow operation pH range, limiting their breadth of application, even though they exhibit excellent antifouling behavior [21]. Therefore, PVDF membranes developed with enhanced hydrophilic properties could address several critical industrial problems.

At present, the ultrafiltration (UF) of a zwitterionic surface membrane has been developed through the “grafting from” approach. However, several grafting methods can be developed to make a specific layer on the supporting membrane, such as UV exposure [22], ozone pre-activation [17], carbon tetrafluoride (CF_4_) plasma-induced graft using PVDF [23], PVDF surface modification of sulfonation [24], plasma-induced polymerization of poly(acrylic acid)-self assumed ZnO [25], pH-dependent thermoresponsive graft poly[2-(diethylamino)ethyl acrylamide] (PDEAEAM) on PVDF surface [26], photo-initiated grafting polymerization [27], electron beam applied surface graft [28], and atom transfer radical polymerization [29]. There are several disadvantages to their large-scale production methods including complex manufacturing steps, high cost, reaction rate, layer formation of <20 nm, and low stability. The main drawback of the PVDF surface is its severe hydrophobicity leading to significant fouling effect on the protein solution. The PVDF membrane covers a wide area, which shrinks during the drying process, and the reduction of porosity is finalized with a uniform pore size of the membrane surface. To develop more useful techniques, the PVDF surface has been modified through UV treatment of interfacial polymerization to improve the chemical and oxidation resistance, and thermal stability [22]. Consequently, the modification to the PVDF membrane surface enhanced the antifouling property of the protein-separation performance.

Several organic/inorganic materials were used for the modification of the commercial PVDF membrane surface by performing UF on the PVDF surface on acrylic and amino monomers using UV photo-grafting [30]. Muchtar et al. reported on a poly(vinylidene fluoride) surface-coated polydopamine using UV light treatment to enhance bovine serum albumin (BSA) rejection [31], PVDF-graphene oxide (GO)/TiO_2_ hybrid membrane was used to enhance BSA performance under UV-light irradiation [32], and TiO_2_/PVDF membrane was treated with UV light to improve the self-cleaning (antifouling) performance of BSA [33]. In addition, UV-treated surface modification of the PVDF membrane gives better performance, including excellent physicochemical absorption and high stability, which enables strong antifouling performance of the membrane, making it a potential candidate for the UF applications.

Unlike polyethylene glycol (PEG) and zwitterion, hydrophilic materials can improve a layer’s hydrophilic properties and forestall protein adsorption or grip on a superficial level or into the pore of the layers [34,35,36]. For instance, the zwitterion material used in this study, sulfobetaine methacrylate (SBMA), has an ammonium moiety (NH^4+^) and a sulfite group (SO^3−^), and they can bond with eight water molecules employing electrostatic cooperation without significant induced stretching [37]. A few common techniques, such as biomimetic bonds [38], joining[17,39], mixing [40], interfacial polymerization [36] and surface throwing [34] can enable the effective preparation of adjusted zwitterion into the layers. In other surface-adjustment strategies, mixing is progressively more viable for layer change for a few reasons: there is no pre-treatment required, it is a straightforward activity, and the resultant film exhibits incredible solidity. In any case, these procedures may make the surface throwing solution become heterogeneous or turbid because of the low dissolvability of the adjusted copolymer that is broken up in the dissolvable [41]. This is because the copolymer (i.e., zwitterion and layer forerunner—PVDF + SBMA), which is integrated before the film arrangement, would become more hydrophilic than the unadulterated film antecedent. Subsequently, this issue might be tackled by employing another technique that includes mixing the zwitterion with a hydrophobic film, such as PVDF, through a connection that has hydrophobic and hydrophilic limits.

In this work, we explored the PVDF surface modification of PSBMA by using simple UV-light irradiation at different time intervals. The physicochemical properties of PVDF/PSBMA modified membranes were investigated to confirm the surface changes using various analytical tools. The improvement of membrane performance and rejection were determined using a model protein foulant (BSA solution). Further investigations were performed to determine the antifouling behavior and regeneration ability of the membrane using a long-term stability test by challenging it in three consecutive cycles. The resulting membrane was then fabricated using eco-friendly large-scale production, which is a promising modification for commercialization.

## 2. Materials and Methods

### 2.1. Materials

Commercial membrane PVDF UF membrane was provided by UlturaTM Inc. (PVDF400, Oceanside, CA, USA). Methanol, ethanol, *N*-(3-Sulfopropyl)-N-methacroyloxyethyl-*N*,*N*-dimethylammonium betaine (SBMA, purity 98%) and phosphate-buffered saline (PBS) tablets were supplied by Sigma–Aldrich (St. Louis, MO, USA). Benzophenone (BP) was obtained from VWR (Atlanta, GA, USA). Bovine serum albumin (BSA, Mw = 66 kD) was received from Lee BioSolutions (Maryland Heights, MO, USA). ACS Reagent grade reagents were used in this study without any purification and post-treatment.

### 2.2. Membrane Modification

**Scheme 1:**
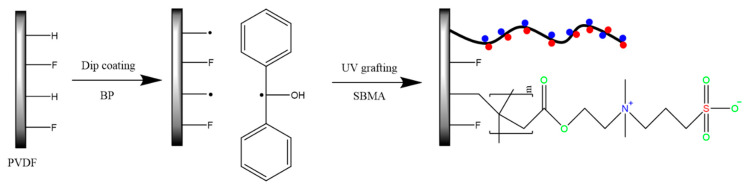
The polyvinylidene difluoride (PVDF) modification of the sulfobetaine methacrylate (SBMA) monomer using the UV treatment polymerization process.

The unmodified PVDF membrane was immersed in a 50% ethanol/water solution for 3 h to eliminate the impurities and glycerin from the membrane. Then, the membrane was kept in a vacuum and oven dried at 40 °C for 24 h to remove moisture and ethanol. To generate a free radical over the membrane surface, the membrane was immersed in 1% BP/methanol used as a photo-initiator for 30 min. Afterwards, the membrane was removed and transferred to the grafting solution (SBMA/DI water) with a certain percentage placed in a petri dish for 30 min of drying under a dark hood to prevent photo-initiation. The UV system (Uvaprint 100) was procured from Honle UV America Inc., MA. The SBMA solution and membrane sample were covered by the quartz petri dish and placed into the UV reaction with a UV lamp intensity of 45 mW/cm^2^ for different time intervals (PSBMA_x_; x: 30 s, 60 s, 120 s, 180 s and 300 s) [42]. After the modification, the residual chemicals in the solution, monomer SBMA, BP, and poly (SBMA) were washed in DI water and methanol. The resulting membrane was kept in a vacuum and oven dried for 24 h to obtain the PVDF-modified PSBMA membrane. The mechanism of the UV-grafting SBMA brush membrane is displayed in Scheme 1.

### 2.3. Characterization of Membranes

The PVDF-modified PSBMA membrane’s chemical composition and the functional group were scrutinized using X-ray photoelectron spectrometry (XPS; Thermo Fisher Scientific Inc., Waltham, MA, USA) and attenuated total reflectance Fourier transform infrared spectroscopy (ATR–FTIR; Perkin Elmer Spectrum 100 FT-IR Spectrometer, Waltham, MA, USA) analysis, respectively. To confirm the surface hydrophilicity of the membrane, we used the water contact angle measurement (WCA; model OCA15EC). The morphology of the PVDF-modified PSBMA membrane was examined by field-emission scanning electron microscope (FE-SEM S-4800) and its surface charge was measured by the zeta potential (SurPASS Electrokinetic Analyzer, Anton Paar, Ashland, VA, USA).

### 2.4. Filtration Performance Studies

Water permeability and rejection performance testing of the membranes were carried during the ultrafiltration operations using a diaphragm pump (P800, King-Kong, Taiwan) with an active membrane area of 12 cm^2^ [36]. All prepared membranes were examined using a stable flux rate at 0.2 MPa for 60 min. Then, the pure water flux was recorded under a pressure of 0.1 MPa for 60 min at 25 °C. BSA solution was introduced to permeate through the membrane. The pure water flux (Jw) and rejection (R) of all membranes were defined as the following Equation [3]:

(1) $$J=\frac{\Delta V}{\left(A\times \Delta t\right)}$$

(2) $$Rejection\;\left(\%\right)=\left(1-\frac{Cp}{Cf}\right)\times 100$$

where, *V* is the volume of permeate water (L), A represents the active permeation area (m^2^), ∆*t* is the permeability time (h), and C*p* and C*f* are the BSA concentration (mg L^−1^) and permeation of the feed solution, respectively.

Dynamic fouling performance studies: In the regular arrangement of a model protein, BSA was utilized for an antifouling experiment. BSA solution at 1000 ppm was examined for antifouling filtration performance. Initially, the pure water flux was measured by using a pressure of 0.2 MPa for 60 min in the steady-state performance. Then, the BSA feed solution was employed at the flux rate (J_p_) at 0.1 MPa for 60 min, while physical washing was used to feed pure water at 0.2 MPa for 30 min and 0.1 MPa for 30 min to remove the foulant from the porous membrane surface.

## 3. Results

### 3.1. Spectroscopy Analysis of the Modified Membrane

**Figure 1:**
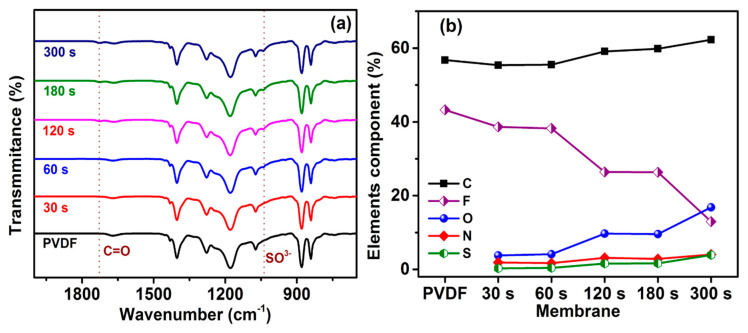
(**a**) Attenuated total reflectance Fourier transform infrared spectroscopy (ATR-FTIR) spectrum of PVDF-modified membrane. (**b**) X-ray photoelectron spectroscopy (XPS) spectrum analysis of the elemental ratio for the PVDF-modified membrane.

The PSBMA of UV treatment timing depends on the surface chemical composition, which was analyzed using ATIR-FTIR spectroscopy. The PVDF membrane surface functional group is compared with a PSBMA layer formation to confirm the new peak absorption in Figure 1a. The new absorption peak at 1726 cm^−1^ exists due to the C=O stretch vibration of PSBMA in the interaction with the membrane surface. The present peak at 1040 cm^−1^ can be attributed to the symmetric stretch vibrations of sulfonate (SO^3−^) groups [41,43]. Thus, FTIR results demonstrate the successful PVDF surface modification of zwitterionic copolymerization (PSBMA). To confirm the successful modification, XPS was used to provide a more detailed analysis.

XPS provides a valuable analysis of the surface chemical composition for the prepared membrane. Figure 1b shows that the unmodified PVDF membrane was observed with stronger peak signals of the C and F ratio compared with a previous report [41]. The UV treatment membrane exhibits new peaks for the O, N, and S ratio. The new peak intensity increases with decreased peak intensity of the F ratio. The ratio of C peaks is varied, with a PSBMA copolymerization on the PVDF surface. After UV treatment was employed, the PSBMA polymerization linearly increases on the PVDF surface to improve the elemental ratio in Table 1. The PSBMA brush chain on the PVDF surface shows that the well-polymerization was successful, as confirmed with an XPS and FTIR, and the modified membrane surface was used for the UF application.

### 3.2. Surface Morphology Analysis

**Figure 2:**
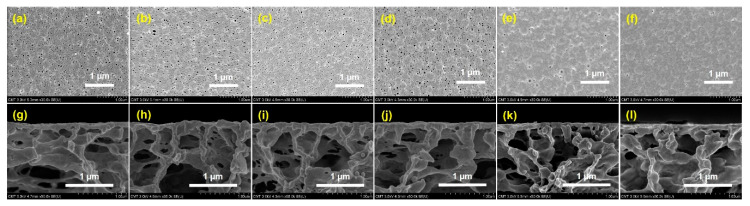
SEM images of surface and cross-section morphology for (**a**,**g**) PVDF, (**b**,**h**) PVDF-PSBMA_30s_, (**c**,**i**) PVDF-PSBMA_60s_, (**d**,**j**) PVDF-PSBM_120s_, (**e**,**k**) PVDF-PSBMA_180s_, and (**f**,**l**) PVDF-PSBMA_300s_.

SEM images were investigated for the membrane surface modification properties of PVDF-SBMA_x_ and unmodified PVDF. In Figure 2a–j, the standard open-pore layers of the membrane surface were observed, and there was a linear increase in the crosslinking grafting ratio of SMBA monomer at different time intervals of polymerization from 30 s to 300 s during UV-light treatment. Then, the modified membrane surface exhibited open-pore crosslinking stability of thin skin-like layer structures, unlike the unmodified PVDF membrane. This could enhance the permeability and rejection of fouling molecules to improve the stability performance of that membrane. The cross-section images of the unmodified PVDF and PSBMA-modified membrane are shown in Figure 2g–l. In the porous supporting PVDF membrane surface, good physical stability was observed, improving the porous substrate during UV-light treatment. Therefore, PSBMA polymerization can be utilized in grafting studies.

### 3.3. The Grafting of PSBMA and Water Contact Angle Studies

**Figure 3:**
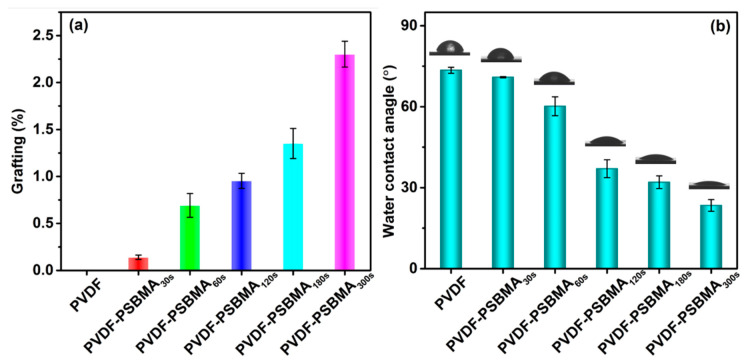
(**a**) The influence of the PVDF membrane modified with SBMA monomer. (**b**) The water contact angle of PVDF and PVDF-modified membrane surface.

Figure 3a shows that the grafting yield of the PSBMA layer at the PVDF membrane surface improved because the UV treatment time rises from 30 to 300 s. SPBMA polymerization of the unmodified PVDF membrane surface was investigated using the degree of grafting method to confirm crosslinking on the membrane following the different time interval treatments. The degree of grafting (DG) yields were estimated in the range of from 0.14% to 2.3% using the following Equation (3).

(3) $$DG\;\left(\%\right)=\frac{W_{1}-W_{0}}{W_{0}}\times 100$$

where, *W*_0_ is the weight of unmodified membrane, and *W*_1_ is the weight after grafting the polymer chain [42]. Polymerization was observed in the membrane surface morphology (Figure 2a–f). Furthermore, the decreased water contact angles (WCA) show that the PVDF-PSBMA membranes’ surface hydrophilicity increases with a growth of PSBMA-grafted polymerization at the PVDF membrane surface. The lowest WCA value of the PVDF-PSBMA_300s_ membrane surface was observed at 23°, which suggests an apparent growth in hydrophilicity when compared with the unmodified PVDF membrane. The WCA changed for grafting yields above 0.14% to 2.3%, which shows the grafting PSBMA polymerization on the PVDF membrane surface.

The surface hydrophilicity behavior is a key factor in filter membrane studies. The PSBMA surface modification membrane was examined using water contact angle (WCA) measurements. In Figure 3b, the WCA value for the unmodified PVDF membrane was 73.5°. Subsequently, the PSBMA solution was treated with a UV light for 0.5 to 5 min, and the initial WCA decreased gradually from 70.93° ± 0.25° to 23.43° ± 2.18° with the increased layer formation of PSBMA. However, the blended membrane surface exhibits a gradual reduction of WCA values to confirm the copolymer modified membrane surface has an exceptional hydrophilicity behavior. There is an important factor that affects the decay rate of WCA, which includes membrane surface charges, initial pore size channels, and wettability of inner pore channels. In our work, for PSBMA on the enclosed surface and internal pore size, there is an effect of WCA changes. When the membrane surface is dribbled with water drops, it easily spreads instantly, owing to the effective hydrogen layer established through the electrostatic interaction between water molecules and the zwitterion’s membrane surface. Compared with a PSBMA membrane, the resultant membrane surface has a faster decay rate of WCA, which is also better than the unmodified PVDF membrane surface. Therefore, the PSBMA-modified membrane surface exhibits excellent hydrophilicity behavior, which improves its antifouling properties.

### 3.4. Zeta Potential of Surface Charge Analysis

**Figure 4:**
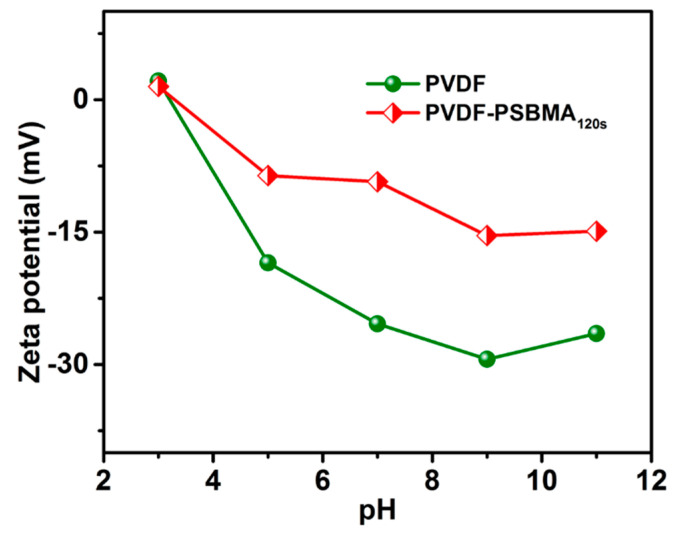
Zeta potential of unmodified PVDF and PVDF-PSBMA_120s_ membranes in different pH solutions.

The zeta potential was measured for the membranes to study the effect of surface charge on the pH or electrolyte solution. Figure 4 shows how the unmodified PVDF and PVDF-PSBMA_120s_ membrane surface charge changed at different pH solutions. The PVDF-PSBMA_120s_ membrane showed a positive charge in the weaker base on a sulfonate group as opposed to an acid medium, and the negative charge shows the sulfonate and carboxylic functional group through the aqueous solution, while the zeta-potential performance was consistent for the modified membrane surface. The unmodified PVDF membrane and PVDF-PSBMA_120s_ membrane exhibit a negative charge in the aqueous solution. The PSBMA_120s_ membrane’s surface charge was observed to enhance hydrophilicity behavior on the SO^3−^ foundation group present in the membrane. Therefore, the unmodified PVDF membrane and PSBMA_120s_ membrane pore surfaces exhibit surface charge properties which enhance antifouling performance.

### 3.5. Pure Water Flux and BSA Feed Water Flux Studies

**Figure 5:**
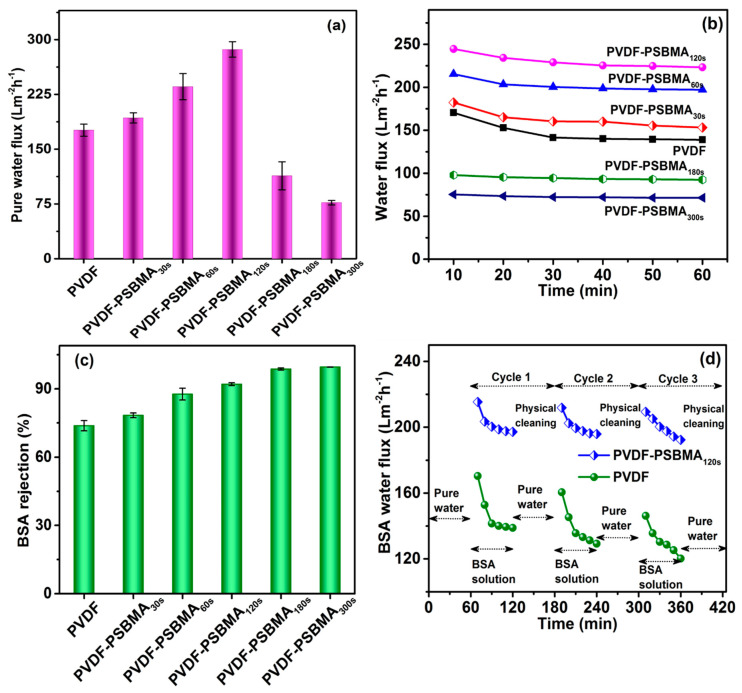
(**a**) The pure water flux of PVDF and PVDF-modified membranes at an applied pressure of 0.1 MPa. (**b**) The water flux of BSA feed solution at a pressure of 1 bar and (**c**) the corresponding rejection performance. (**d**) The antifouling performance of PVDF and PVDF/PSBMA membranes for three cycles in the BSA feed solution at a pressure of 0.1 MPa.

During the PSBMA interaction with a PVDF membrane surface we measured the pure water flux performance by using the UF operation mode. In Figure 5a, the pure water flux of the unmodified PVDF membrane was ≈176 Lm^−2^h^−1^. When the PSBMA UV treatment time increased, the pure water flux values were 192, 235, 286, 113, and 76 L m^−2^h^−1^ as the interval went from 30 s to 300 s, respectively. The water flux exhibited the ability of surface modification of the PSBMA layer formation to improve surface properties and the stability of the membrane (Figure 2; SEM images). For the optimum UV treatment time of 120 s, we observed good hydrophilicity and more-stable pore interactions after polymerization of PSBMA, which in turn enhanced water permeability. However, UV treatment times shorter than 120 s were unable to form a tight-enough hydration layer on the membrane owing to a lack of zwitterionic moieties. The UV exposure time longer than 120 s exhibited a significant enhancement in surface hydrophilicity, as demonstrated by contact angle measurement results (Figure 3b). Moreover, as the surface hydrophilicity was improved, the mass transport resistance also gradually increased as the excess of polymer chain grew on the membrane, as evidenced in Figure 2 (SEM image). The surplus amount of poly-SBMA blocked the pore with longer UV exposure times of 180 s and 300 s. Therefore, the most appropriate and optimum UV exposure time of 120 s was determined from both chemical and physical aspects to achieve highest water flux.

To investigate antifouling resistance performance of the unmodified PVDF and PSBMA-modified surface membrane, water flux studies were carried out using BSA as the model fouling solution. In Figure 5b, it can be seen that the unmodified PVDF membrane performance greatly decreased from 170.4 to 138.9 Lm^−2^h^−1^ of the BSA feed water flux up to 60 min, while BSA rejection performance was more stable at 73.79% ± 2.24% during the continued feed solution time of 60 min. The PSBMA_120_s modified membranes showed a significantly decreased BSA feed water flux value from 245 to 223 Lm^−2^h^−1^; this result was better than the PSBMA-modified membrane with a low or high duration of UV-light treatment, which had a weak pore stability, and the skin layer formed at the surface decreased the BSA feed water flux value. BSA rejection was slightly variable, with 78.3%, 87.6%, 98.7% and 99.6% for PSBMA_30s_, PSBMA_60s_, PSBMA_180_s and PSBMA_300s_, respectively. All surface-modified membranes showed better permeability and rejection compared with the unmodified PVDF membrane. Figure 5c shows that the BSA rejection reached 99.6% as the UV-light treatment time increased, but we consider that the high BSA feed water flux is optimized for further antifouling studies. Moreover, the remarkable increase in the permeability of the PSBMA membrane exhibits a uniform pore size and good hydrophilicity, which improves the zwitterionic membrane performance by a 2–3-fold increment compared with the previously reported PVDF-modified membranes [39].

### 3.6. Dynamic Antifouling Property Studies

The antifouling properties of the PSBMA-modified membrane polymerization were demonstrated using a model protein foulant, BSA, and the influence of PSBMA crosslinking stability was investigated. Considering the robust interlayer permeability of the water molecules in the poly-zwitterions, the PSBMA modified membrane exhibits good antifouling properties. Figure 5d shows the time-dependent BSA feed water flux of the unmodified PVDF and PVDF-PSBMA_120s_ membranes. During the first 60 min of the pure water stability process of ultrafiltration, the flux rate of the membrane slowly decreased for unmodified PVDF and PVDF-PSBMA_120s,_ with an unstable increase in porosity and pore size. Then, the flux rate of BSA solution reduced rapidly at the preliminary stage, due to protein fouling and concentration polarization [44]. However, the concentration polarization exhibits the electro repulsion on the membrane surface, which is related to the hydrophilicity behavior in these studies. The flux rate slightly decreased the main role of protein fouling on the surface, and the small amounts of BSA were close to the pore size. The PVDF-PSBMA_120s_ membrane performed more improved BSA feed water fluxes than the unmodified PVDF membrane during the three-cycle experiments. When the flux rate increased, there was undoubtedly an improvement of hydrophilicity because the hydrophilic membrane was more stable, and the antifouling ability was good. A greater recovered water flux has been acquired from grafted PSBMA brush and stable pore size membrane features for up to three cycles after the cleaning technique process; the more zwitterionic the membrane surface, the more stable the flux rate. In addition, the water flux recovery ratio (FRR) and BSA rejection ratio were analyzed: The first and third cycle of PSBMA-grafted membrane had better water permeability than the unmodified PVDF membrane. The hydrophilicity and antifouling characteristics demonstrate the effective development of this PSBMA polymer after zwitterionic grafting on the surface and within the pores of the unmodified PVDF membrane.

## 4. Conclusions

In this work, a zwitterionic polymer was grown on a commercial PVDF membrane using the “grafting from” surface modification method. The ultrafiltration performance and antifouling properties of the modified-surface membrane were investigated in detail. The SBMA monomer was polymerized on top of a PVDF membrane surface through a photo-initiator (BP) coating and the UV-grafting method. The degree of grafting and physicochemical properties were systematically investigated in detail. The degree of grafting was gradually enhanced following an irradiation time interval increase, which was evidenced by the XPS analysis and surface morphology. Rejection was promoted at an increased irradiation time and this led to the enhancement of water transport resistance. Consequently, the optimized modified membrane performance was evaluated, with an increased pure water flux value ~66% that of the unmodified PVDF membrane. Additionally, the antifouling properties of the PVDF-PSBMA_120s_ membrane were evaluated using a model foulant BSA rejection and up to a three-cycle test. The outstanding antifouling properties were attributed to the hydrophilic membrane surface and hydration layer with a zwitterionic polymer brush. Overall, these results exhibited excellent antifouling properties, which effectively improved the use of PSBMA UV-grafting polymerization on the membrane surface; hence, the reported surface modification step is promising in terms of future commercial applications.

## Figures and Tables

**Table 1:** The XPS spectrum was evaluated using the elemental ratio of the unmodified PVDF and PSBMA-modified membrane.

Membrane	C (%)	F (%)	O (%)	N (%)	S (%)
PVDF	56.76	43.24	-	-	-
30s	55.38	38.63	3.81	1.87	0.31
60s	55.52	38.24	4.14	1.7	0.4
120s	59.12	26.43	9.7	3.16	1.59
180s	59.53	26.37	9.6	2.85	1.65
300s	62.25	12.97	16.82	4.06	3.9
